# 

**Published:** 1906-09

**Authors:** 


### BOOKS RECEIVED.

Medical Jurisprudence. Forsenic Medicine and Toxicology. By R. A. Witthaus, A.M., M.D., Professor of Chemistry, Physics and Toxicology in Cornell University; and Tracy C. Becker, A.B., LL. B. Professor of Criminal Law and Medical Jurisprudence in the University of Buffalo. Vol. I. Octavo, pp. LV-996. Second edition, New York: William Wood & Co. 1906. (Muslin, $6.00; Law sheep, $7.00, net prices).

The Chemistry, Physiology, and Pathology of Uric Acid. With a discussion of the Metabolism in Gout. By Francis H. McCrudden, of the Laboratory of Physiological Chemistry, Harvard Medical School.

Proceedings of the American Medico-Psychological Association at the sixty-first annual meeting held at San Antonio, Texas, April 18-21, 1905. Charles W. Pilgrim, M.D., Secretary elect.

Eczema. A consideration of its course, diagnosis and treatment, with 146 prescriptions. By Samuel Horton Brown, M.D., Assistant Dermatologist in the Philadelphia Hospital. 12 mo. pp. 105. Philadelphia: P. Blakiston's Son & Co. 1906. (Price $1.00).

A Treatise on Surgery. In two volumes. By George R. Fowler, M.D., Examiner in Surgery, Board of Medical Examiners of the Regents of the University of the State of New York; Emeritus Professor of Surgery in the New York Polyclinic, etc. Two imperial octavos of 725 pages each, with 888 text illustrations and 4 colored plates, all original. Philadelphia and London: W. B. Saunders Company. 1906. (Per set: cloth, $15.00, net; half-morocco, $17.00, net).

Progressive Medicine, Vol. II, June, 1906. A Quarterly Digest of Advances, Discoveries and Improvements in the Medical and Surgical Sciences. Edited by Hobart Amory Hare, M.D., Professor of Therapeutics and Materia Medica in the Jefferson Medical College of Philadelphia. Octavo, 368 pages, 31 illustrations. Lea Brothers & Co., Philadelphia and New York. (Per annum, in four cloth-bound volumes, $9.00; in paper binding, $6.00; carriage paid to any address).

Surgical Suggestions. Practical Brevities in Surgical Diagnosis and Treatment. By Walter M. Brickner, M.D., Chief of Surgical Department, Mount Sinai Hospital Dispensary, New York; Editor American Journal of Surgery, and Eli Moschowitz, M.D., Assistant Physician, Mount Sinai Hospital Dispensary, New York. Duodecimo; 60 pages. New York: Surgery Publishing Co. 1906. (Cloth, 50 cents).

A Compend of Operative Gynecology. Based on Lectures in the Course of Operative Gynecology on the Cadaver. Delivered by William Seaman Bainbridge, M.D., Adjunct Professor of Operative Gynecology on the Cadaver, New York Post-Graduate Medical School and Hospital. In Collaboration with Harold D. Meeker, M.D., Instructor in Operative Gynecology on the Cadaver, New York Post-Graduate Medical School and Hospital. 12mo, 76 pages. The Grafton Press, New York. (Price, $1.00).

The Practice of Pediatrics by American and English Authors. Edited by Walter Lester Carr, M.D., Consulting Physician to the French Hospital; Visiting Physician to the Infants' and Children's Hospital, New York. In one octavo volume of 1014 pages with 199 engravings and 32 full page plates in colors and monochrome. Lea Brothers & Co., Philadelphia and New York. 1906. (Cloth, $6.00; leather, $7.00; half morocco, $8.00, net prices).

Clinical Diagnosis. A Textbook of Clinical Microscopy and Clinical Chemistry. By Charles Phillips Emerson, A.B., M.D., Resident Physician in John Hopkins Hospital; Associate in Medicine in the John Hopkins University. Octavo, pp. 661. Illustrated. Philadelphia: J. B. Lippincott Co. 1906.

The Prophylaxis and Treatment of Internal Diseases. By F. Forchheimer, M.D., Professor of Theory and Practice of Medicine and Clinical Medicine in the Medical College of Ohio (University of Cincinnati.) Octavo, pp. 669. New York and London: D. Appleton & Co. 1906. (Price, $5.00, net).

Report of the Commissioner of Education for the year ending June 30, 1904. In two volumes. Washington: Government Printing Office. 1906.

Blakiston's Quiz Compends. Materia Medica, Therapeutics and Prescription Writing. By Samuel O. L. Potter, M. D., formerly Professor of the Principles and Practice of Medicine in the Cooper Medical College of San Francisco. Seventh edition, revised and enlarged. Philadelphia: P. Blakiston's Son & Co. 1906. (Price, $1.00).

A Nonsurgical Treatise on the Diseases of the Prostate Gland and Adnexa. By George Whitfield Overall, A.B., M.D. 12mo, pp, 228, Illustrated. Rowe Publishing Co. 1906.

International Clinics. A quarterly of Illustrated Clinical Lectures and especially prepared articles on Treatment, Medicine, Surgery, Neurology, Pediatrics, Obstetrics, Gynecology, Orthopedics, Pathology, Dermatology, Ophthalmology, Otology, Rhinology, Laryngology, Hygiene and other topics of interest to students and practitioners. By leading members of the medical profession throughout the world. Edited by A. O. J. Kelly, A.M., M.D., Philadelphia. Volume II. Sixteenth series. 1905. Philadelphia and London: J. B. Lippincott Co. (Cloth, $2.00).

